# Role of Brain Networks in Burning Mouth Syndrome: A Narrative Review

**DOI:** 10.3390/dj13070304

**Published:** 2025-07-04

**Authors:** Takahiko Nagamine

**Affiliations:** 1 Department of Psychiatric Internal Medicine, Sunlight Brain Research Center, Hofu 747-0066, Japan; anagamine@yahoo.co.jp; Tel.: +81-835-25-6610; 2 Graduate School of Medical and Dental Sciences, Institute of Science Tokyo, Bunkyou 113-8510, Japan

**Keywords:** brain networks, burning mouth syndrome, dopamine, functional magnetic resonance imaging, medial pain system, medial prefrontal cortex

## Abstract

**Objective**: Burning mouth syndrome (BMS) is a chronic and often debilitating orofacial pain condition characterized by a burning sensation in the oral mucosa without clear abnormal lesions. While its etiology is considered multifactorial, the underlying pathophysiology remains unclear. This narrative review aims to synthesize existing functional magnetic resonance imaging (fMRI) studies to shed light on the central neural mechanisms contributing to BMS. **Methods**: A focused electronic search was conducted across the PubMed and J-STAGE databases for relevant articles published in English from January 2000 to May 2025. The review prioritized studies investigating brain structure and function using fMRI in individuals with BMS. **Results**: Our synthesis of the literature consistently demonstrated that the brains of individuals with BMS exhibit augmented connectivity within the medial pain system and a diminished gray matter volume in the medial prefrontal cortex (mPFC). These findings suggest a crucial role for altered brain circuitry, particularly a reduction in the output of the basal ganglia dopamine system, in the experience of BMS pain. **Conclusions**: The consistent fMRI findings strongly indicate that BMS involves significant functional and structural brain alterations. The observed changes in the mPFC and its connections to the basal ganglia dopamine system highlight this pathway as a potential target for both pharmacological and non-pharmacological neurological interventions for individuals with BMS.

## 1. Introduction

Burning mouth syndrome (BMS) is a challenging and often debilitating chronic orofacial pain syndrome, characterized by a persistent burning sensation in the oral mucosa without any clear physical lesions. Individuals with BMS endure constant or intermittent burning pain of unknown origin, which can last for months or even years, significantly diminishing their quality of life [1]. This condition occurs disproportionately in women, with an average age of onset typically in the perimenopausal or postmenopausal period. While the precise cause of BMS remains elusive, it is understood to be a complex interplay of factors, including neuropathic pain, hormonal changes, nutritional deficiencies, and psychological elements [2]. The International Classification of Orofacial Pain (ICOP) categorizes BMS as an idiopathic orofacial pain, notably lacking overt morphological alterations or evident nerve damage in the oral cavity [3].

Given the absence of detectable oral lesions, it has been increasingly speculated that the pain experienced in BMS may stem from disruptions within the brain’s pain-related circuits, including the pain matrix [4]. To gain deeper insight into these central mechanisms, this narrative review was conducted to comprehensively examine and synthesize the existing literature utilizing functional magnetic resonance imaging (fMRI) to explore brain structure and function in individuals with BMS. Our analysis aims to present a cohesive understanding of the neural alterations underlying this perplexing condition, aligning with the growing recognition of BMS as a centrally driven, nociplastic pain syndrome.

## 2. Materials and Methods

The aim of this study was to investigate what neural network changes identified by fMRI studies in adults diagnosed with BMS compared with healthy controls are associated with the pathological mechanisms of BMS (Table 1).

To provide a comprehensive overview and synthesize key insights into the relationship between BMS and neural circuits, a narrative review was conducted. Rather than systematically mapping all available evidence, this approach focused on identifying and interpreting influential studies that shed light on brain structure and function in BMS patients, particularly those utilizing fMRI.

A targeted search for the pertinent literature was performed across the PubMed and J-STAGE databases, encompassing articles published in English from January 2000 to May 2025. The search strategy employed keywords such as burning mouth syndrome, neural circuits, functional magnetic resonance imaging, and mechanism, combined with Boolean operators to identify highly relevant studies. We prioritized original articles and conference proceedings that included fMRI data, such as resting-state fMRI, task-related fMRI, and diffusion tensor imaging (DTI), from patients diagnosed with BMS according to international criteria. Studies that compared BMS patients with control groups or examined differences within BMS patient populations were of particular interest. Exclusions included case reports, review articles, animal studies, and those employing brain imaging techniques other than fMRI. Studies focusing exclusively on secondary BMS or involving patients with fMRI contraindications were also not included. The focus was on gathering a rich set of information to construct a cohesive narrative, rather than on a formal assessment of individual study quality or risk of bias.

## 3. Results

### 3.1. Unraveling Brain Changes in Burning Mouth Syndrome

**Figure 1:**
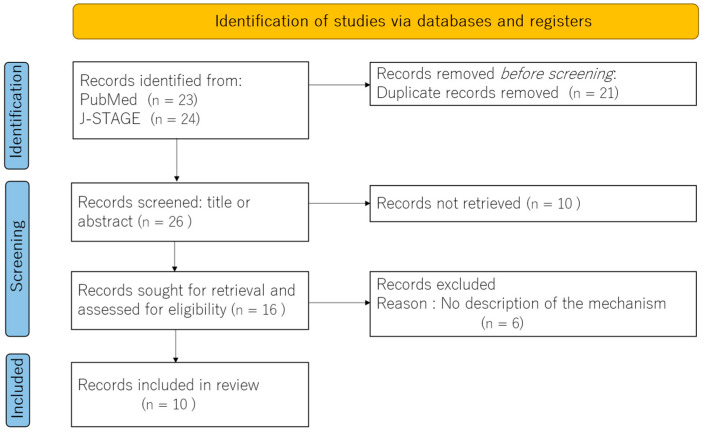
A PRISMA flow diagram.

Our exploration of the literature aimed to understand the neural underpinnings of burning mouth syndrome (BMS) through fMRI studies. From an initial broad search, ten key papers emerged as particularly relevant, providing critical insights into how the brains of individuals with BMS differ from those of healthy controls. A PRISMA flow diagram is shown in Figure 1. These studies consistently revealed differences in neuronal connectivity and altered brain activity within regions crucial for pain perception and emotional processing in BMS patients. To better understand these structural and functional brain changes, we categorized the identified literature into two main approaches: those employing sensory stimulation during fMRI and those examining resting-state brain activity without external stimuli.

### 3.2. Brain Responses to Sensory Stimulation

Four of the included studies utilized fMRI while presenting specific sensory stimuli, offering a window into how BMS brains react to various inputs. These stimuli included noxious thermal stimulation to the lower lip [5,6], separate thermal stimulation to the right palm and right lower lip [7], and even tactile stimulation combined with the presentation of angry faces [8]. Across all these stimulated fMRI studies, individuals with BMS consistently showed heightened activation in neural regions comprising the “pain matrix” when compared to healthy controls.

Specifically, applying thermal stimuli to the trigeminal nerve area (which supplies sensation to the face) led to significantly greater activation in key areas like the medial prefrontal cortex (mPFC), the anterior cingulate cortex (ACC), the insular cortex (IC), and the hippocampus (Hc) in BMS patients [5,7]. For example, noxious thermal stimulation to the lower lip notably increased activation in the ACC and para-hippocampal gyrus [6]. Similarly, when gradually increasing painful thermal stimuli were applied to either the hand or lower lip, BMS brains demonstrated substantial sensitivity to these temperature changes, with the mPFC, IC, and ACC showing significantly elevated activity [7]. Even tactile stimuli, when an emotional state was induced, elicited a heightened sensitivity in individuals with BMS [8]. These findings are summarized in Table 2, providing a closer look at how BMS brains react under sensory challenge.

### 3.3. Intrinsic Brain Activity and Structural Changes

The majority of the studies, six in total, investigated brain structure and function in BMS without external stimulation, allowing us to examine baseline brain activity and intrinsic networks when the patient is at rest. Although these studies used varied methodologies for analyzing fMRI data (making direct meta-analysis challenging), they yielded comparable and compelling results, consistently pointing to significant alterations in BMS brains.

A recurring finding across these non-stimulated fMRI studies was a reduction in gray matter volume in the mPFC and robust connectivity between the mPFC and the amygdala (AMY) in individuals with BMS. One study using resting-state fMRI specifically noted an increased gray matter volume in the Hc alongside a marked decrease in the mPFC in BMS patients compared to controls [9]. Another study, employing voxel-based morphometry, found a correlation between gray matter density in the pain matrix (including the ACC, cerebellar lobule, inferior temporal lobe, and mPFC) and pain intensity specifically in BMS individuals, a correlation absent in healthy controls and those with dysgeusia [10]. Gray matter density itself reflects the concentration of neural cells and their organization within a region.

Further insights into white matter structure and connectivity revealed that BMS brains exhibit significantly stronger connectivity within the medial pain system, as shown by probabilistic tractography. Interestingly, no significant differences were observed in the lateral pain system, which includes the somatosensory cortex [11]. The functional connectivity between the mPFC and the AMY in BMS patients was also found to be correlated with both the duration of the disease and the severity of symptoms [12]. A graph analysis of structural connectivity reinforced these findings, indicating stronger connectivity between regions of the medial pain system (such as the IC, AMY, and mPFC) when comparing BMS individuals to healthy controls [13]. Finally, evidence of astrocytic hypertrophy and microstructural alterations in the AMY of BMS patients points towards underlying functional and structural changes within the emotional processing system [14]. These six studies, focusing on non-stimulated fMRI, are summarized in Table 3, illustrating the complex baseline alterations in BMS brains.

## 4. Discussion

### 4.1. Brain Alterations in Burning Mouth Syndrome

Our narrative review highlights that the perplexing experience of BMS likely stems from a complex interplay of factors, with emerging fMRI evidence strongly pointing to significant functional and structural alterations within the central nervous system. While peripheral small fiber neuropathy and hormonal shifts (particularly in postmenopausal women) are increasingly recognized as contributing elements, the central nervous system’s role in amplifying and perpetuating pain is paramount [15]. BMS can be understood as a form of central sensitization, where the brain becomes hypersensitive to sensory input, leading to amplified pain signals even without clear peripheral damage [16]. This maladaptive neuroplasticity, where the brain reorganizes in ways that reinforce pain perception, along with potential neurotransmitter imbalances, offers a compelling framework for understanding the chronic nature of BMS pain [17]. Functional and structural brain imaging, especially fMRI and DTI, provides invaluable insight into these neural mechanisms, revealing areas of altered activity and structural integrity. Our principal findings from the reviewed fMRI literature can be broadly categorized into functional impairment and structural abnormalities within the brains of individuals with BMS.

### 4.2. Functional Impairment: Reconfigured Pain and Emotional Networks

**Figure 2:**
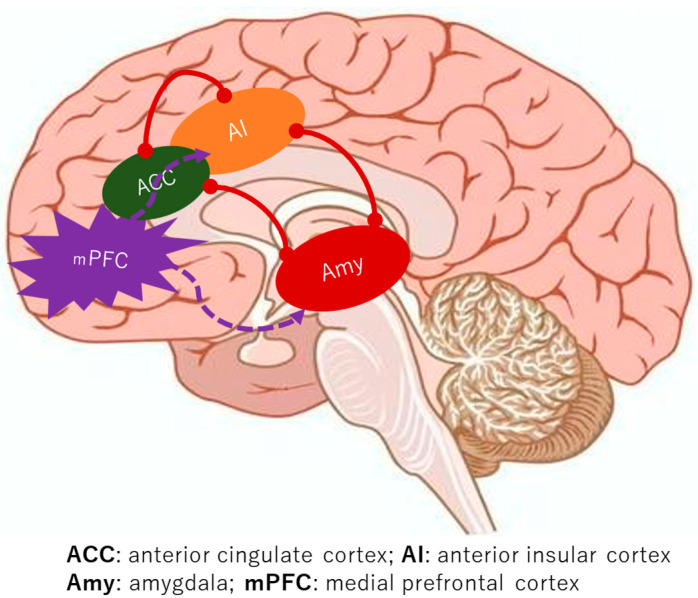
The alterations in neural connectivity in individuals with BMS.

A consistent finding across the studies is the augmented functional connectivity within the medial pain system in BMS patients, encompassing key regions such as the mPFC, ACC, IC, Hc, and AMY [9,10,11,12,13,14]. Notably, the lateral pain system, including the somatosensory cortex, did not show similar discrepancies when compared to healthy controls [5,6,7]. The brain’s intricate functional networks, which govern everything from sensation to emotion, appear to be significantly reconfigured in BMS. A critical aspect of this re-wiring involves the salience network, a key functional brain network formed by the connectivity between the ACC and the IC [18]. This network is crucial for detecting and directing attention towards both external stimuli and internal bodily changes. In BMS, the robust functional connectivity of the salience network, particularly with emotional processing areas like the AMY, suggests a heightened sensitivity to internal signals (Figure 2). This heightened connectivity may lead to the perception of innocuous stimuli as significant changes, potentially triggering or intensifying the experience of pain. This characteristic of enhanced salience network activity and its strong connections to emotional circuits is also observed in other chronic pain conditions like fibromyalgia, hinting at a common neural signature for such disorders [19]. Furthermore, the strengthened connectivity between the salience network and memory-related regions, such as the Hc, offers a compelling explanation for the persistence of chronic pain even in the absence of ongoing noxious stimuli. It is plausible that past oral habits or painful dental procedures could have irritated trigeminal nerves, serving as an initial trigger [20]. The brain, particularly the IC, appears to store “molecular memories” of painful inflammation, and activation of the salience network might trigger the replay of these past painful experiences [21]. This suggests a mechanism where previous pain experiences alter neural connectivity, creating a robust circuit between the salience network, the AMY, and the Hc, thereby facilitating the regeneration of pain sensations.

The brain’s intricate functional networks that regulate pain sensation and emotion undergo a profound reconfiguration in BMS. A fundamental element of this reconfiguration is the tight coupling of the mPFC and the emotional system.

### 4.3. Structural Abnormalities: Altered Brain Architecture

Beyond functional shifts, our review also highlights significant structural abnormalities in the brains of individuals with BMS. These include a reduction in gray matter volume in the mPFC, an increase in gray matter volume in the Hc, and an increase in astrocyte volumes in the AMY [12,14]. Gray matter volume, reflecting the overall size and cellular composition of a brain region, provides vital clues about the underlying pathology. The observed reduction in mPFC volume is particularly significant, given its pivotal role in pain perception and emotional regulation [22]. This decrease could contribute to heightened pain perception and a diminished capacity for pain tolerance in BMS patients. While the precise mechanism behind this volume reduction in chronic pain remains unclear, chronic stress associated with pain and imbalances in neurotransmitters like glutamate and GABA are plausible contributors. The mPFC’s close relationship with the basal ganglia, a region rich in dopamine-secreting neurons involved in pain inhibition, adds another layer of complexity [23]. A reduction in mPFC neurons and impaired function of dopaminergic neurons in the basal ganglia are interconnected and may reduce the brain’s natural analgesic effects, although the causal relationship is unclear [23]. This notion is supported by PET studies showing reduced dopamine D2 receptor activity and diminished endogenous dopamine levels in the striatum of BMS patients. Intriguingly, similar mPFC volume reductions and dopamine dysfunction have been observed in other chronic pain disorders of unknown etiology, suggesting a potential common neurobiological pathway [24]. The increased gray matter volume in the Hc in BMS patients presents a more nuanced finding, as the literature on hippocampal volume in chronic pain is often contradictory [25]. While some chronic pain conditions show hippocampal atrophy linked to memory and sleep issues, the increase observed in BMS (and in conditions like vestibulodynia) might reflect increased information processing, potentially driven by the overactive salience network [26]. Finally, structural alterations in the AMY, including astrocytic hypertrophy and microstructural changes, also appear central to BMS pathogenesis [9,14]. Animal models show that intense painful stimulation can alter AMY neurons, leading to ectopic pain without new stimuli [27]. This suggests that a history of oral procedures, frequently reported by BMS patients, might induce structural changes in the AMY, thereby lowering the pain threshold and contributing to BMS development.

In essence, both resting-state and stimulation-based fMRI provide compelling evidence that the intrinsic brain activity and neural responses to stimuli in individuals with BMS differ profoundly from those in healthy individuals. These studies collectively underscore the involvement of neural circuits, particularly those encompassing the ACC and mPFC, highlighting the significant emotional and cognitive components of BMS. This body of work reframes BMS not as a purely peripheral issue but as a nociplastic pain condition, characterized by an alteration in the pain-processing pathway within the central nervous system, even in the absence of overt tissue or nerve damage. The pain system itself undergoes hypersensitivity, leading to widespread and diffuse pain, often accompanied by fatigue, sleep disturbances, and mood changes, similar to conditions like fibromyalgia. Understanding BMS through this lens is crucial for developing more effective, centrally targeted therapeutic strategies.

## 5. Limitations

While this narrative review aimed to provide a comprehensive and interpretive synthesis of fMRI findings in burning mouth syndrome (BMS), it is important to acknowledge several limitations inherent to this methodological approach and the scope of our investigation.

Firstly, as a narrative review, this study does not adhere to the rigorous systematic protocols of a systematic review or meta-analysis. Consequently, there was no formal assessment of the methodological quality or risk of bias for individual studies. For data such as gray matter volume, due to different measurement methods and limited case numbers, it was not possible to enter these data into the Cochrane Review Manager software (RevMan 5.0) and synthesize them for review using Cochrane MetaView software. Our approach prioritized the comprehensive interpretation and synthesis of key concepts and findings over an exhaustive, protocol-driven aggregation of evidence. This means that while we aimed for a balanced overview, the depth of critical appraisal for each included paper was not as exhaustive as in systematic reviews, and the potential for selective reporting or subjective interpretation is acknowledged.

Secondly, this review serves as a pilot or proof-of-concept study to explore the feasibility and initial landscape of research in brain networks of individuals with BMS. As such, we intentionally limited our search to PubMed and J-STAGE to quickly identify key themes and gaps, with the intention of conducting a more exhaustive multi-database search in a subsequent, full-scale review. This limited scope means that relevant studies published in other databases (such as Web of Science, Embase, or the Cochrane Library), or in languages other than English, may have been inadvertently excluded. Future research employing more exhaustive and diverse search strategies across a wider array of databases would undoubtedly provide an even more comprehensive understanding of the topic.

Finally, while fMRI offers invaluable insights into the neural mechanisms of BMS by directly measuring brain activity and revealing involved regions and their connections in pain processing, it is crucial to recognize that fMRI measures brain activity, not pain itself. The observed brain activity changes must be interpreted in conjunction with other indices that directly evaluate pain, such as patient-reported outcomes or quantitative sensory testing. The complexity of pain perception means that while fMRI data strongly implicate altered brain function in BMS, further research integrating multiple assessment modalities is essential for a complete understanding of the patient’s lived experience of pain.

## 6. Conclusions

This narrative review synthesizes compelling fMRI evidence, demonstrating that burning mouth syndrome (BMS) is characterized by distinct structural and functional alterations within the brain’s pain and emotional processing networks. Our exploration of both stimulated and resting-state fMRI studies consistently reveals that the intrinsic brain activity and neural responses to stimuli in individuals with BMS diverge significantly from those of healthy controls. A central finding is the heightened connectivity within the salience network (comprising the ACC and IC) in BMS patients, and its strong connections to the AMY, Hc, and mPFC. This increased connectivity likely contributes to the persistent experience of pain, even in the absence of obvious external stimuli, by potentially amplifying the perception of internal bodily signals. Furthermore, we consistently observed a reduction in gray matter volume in the mPFC, which appears linked to diminished dopamine function in the basal ganglia. This structural change and its impact on the brain’s pain matrix suggest that the dopaminergic nervous system represents a promising avenue for future therapeutic interventions in BMS.

In essence, our narrative review underscores that BMS is not merely a peripheral discomfort but a complex condition rooted in demonstrable changes in brain structure and function. Understanding these central nervous system alterations is crucial for developing more effective, targeted treatments for individuals suffering from this challenging pain syndrome.

## Figures and Tables

**Table 1:** The PICO of this study.

P (Population)	Adults diagnosed with burning mouth syndrome
I (Intervention)	Studies utilizing functional magnetic resonance imaging (fMRI) to examine neural network activity
C (Comparison)	Healthy controls
O (Outcome)	Identification and description of altered neural network activity and associated brain regions implicated in the pathological mechanisms of burning mouth syndrome

**Table 2:** Results of the fMRI studies under specific stimuli.

Author (Year)	Sample Size	Study Method	Outcome Assessment	Conclusions
Albuquerque et al., 2006 [5]	BMS group: *n* = 8 (female: 8), mean age 49.1 y.o. Control group: *n* = 8 (female: 8), healthy subjects, mean age 50.3 y.o.	fMRI performed while applying thermal stimulation to the trigeminal nerve	In the BMS group, greater regional signal changes were observed in the right anterior cingulate cortex and bilateral precuneus during thermal stimulation of the trigeminal nerve.	The BMS group exhibited brain activation patterns analogous to those observed in patients with other types of chronic neuropathic pain disorders, with danger-detecting circuits, such as the anterior cingulate cortex, demonstrating heightened activation.
Shinozaki et al., 2016 [6]	BMS group: *n* = 16 (female: 16), postmenopausal/perimenopausal, under 65 years Control group: *n* = 15 (female: 15), healthy subjects, under 65 years	fMRI during repetition of noxious heat stimulus on the lower lip using Statistical Parametric Mapping 8 software	In the BMS group, activity in the anterior cingulate cortex and the para-hippocampal gyrus was connected compared to the control group.	In the BMS group, thermal stimulation of the lower lip resulted in the activation of the anterior cingulate cortex and its associated neural systems. This activation was achieved through input from the trigeminal nerve to the central nervous system.
Yoshino et al., 2017 [8]	BMS group: *n* = 27 (female: 21), mean age 44.8 y.o. Control group: 21 (female: 18), healthy subjects, mean age 46.3 y.o.	fMRI when subjects were shown pictures of angry and neutral faces to elicit emotions and were given tactile stimulation	Changes in touch-related activation in the postcentral gyrus evoked by angry facial expressions were significantly positively correlated with pain intensity in daily life in the BMS group.	In the BMS group, neural responses in the postcentral gyrus exhibited heightened sensitivity to angry facial expressions when compared with those observed in healthy subjects. This finding suggests the presence of an association between emotion and pain perception.
Kohashi et al., 2020 [7]	BMS group: *n* = 15 (female: 15), mean age 52.6 y.o. Control group: *n* = 15 (female: 15), healthy subjects, 49.0 y.o.	fMRI with continuous thermal stimulation	In the BMS group, the subjects responded very sensitively to stimulation of the trigeminal nerve, with the medial frontal cortex, insular cortex, and anterior cingulate cortex becoming significantly more activated than in healthy subjects.	The BMS group responded extremely sensitively to pain information generated by the trigeminal nervous system and showed coordinated activation of the so-called salience network and the medial frontal cortex.

Abbreviations: BMS: burning mouth syndrome; fMRI: functional magnetic resonance imaging; y.o.: years old.

**Table 3:** Results of the fMRI studies without stimuli.

Author (Year)	Sample Size	Study Method	Outcome Assessment	Conclusions
Khan et al., 2014 [9]	BMS group: *n* = 9 (female: 9), postmenopausal/perimenopausal Control group: *n* = 9 (female: 9), matched healthy subjects, postmenopausal/perimenopausal	Functional connectivity in resting-state fMRI using voxel-based morphometry and diffusion tensor imaging.	The BMS group exhibited an increase in gray matter volume in the hippocampus, accompanied by a decrease in gray matter volume in the medial prefrontal cortex.	A series of structural alterations were identified in the medial prefrontal cortex and the hippocampus. Concurrently, modifications in brain networks were also observed. These structural changes may have a role in the pathophysiology of mood and depressive symptoms.
Sinding et al., 2016 [10]	BMS group: *n* = 12 (female: 7), mean age 59.4 y.o. Control group 1: *n* = 17 (female:11), dysgeusic patients, mean age 58.4 y.o. Control group 2: *n* = 13 (female:10), healthy subjects, mean age 59 y.o.	Functional connectivity in fMRI using voxel-based morphometry.	In the BMS group, the severity of pain was found to be associated with alterations in gray matter density within several regions of the brain, including the anterior cingulate cortex, the cerebellar lobule, the inferior temporal lobe, and the prefrontal cortex.	A potential underlying factor in the development of BMS is the presence of inadequate pain management. This phenomenon is further compounded by the absence of a correlation between BMS and dysgeusia, suggesting that these conditions may not be attributable to analogous structural alterations within the brain.
Wada et al., 2017 [11]	BMS group: *n* = 14 (female: 14), mean age 50.9 y.o. Control group: *n* = 14 (female: 50.2 y.o.	Structural connectivity was calculated. The brain network of BMS brains was studied by using probabilistic tractography and graph analysis.	A substantial discrepancy in local brain connectivity was identified in the anterior cingulate cortex, medial orbitofrontal cortex, and pars orbitalis of the brain, which are components of the medial pain system. Conversely, no substantial discrepancy was identified in the lateral pain system, encompassing the somatic sensory cortex.	A structural brain network analysis was conducted, revealing alterations in the medial pain system of the pain-related brain network in patients with BMS. These alterations were found to be analogous to those observed in chronic pain patients.
Tan et al., 2019 [12]	BMS group: *n* = 26 (female: 21), mean age 52.12 y.o. Control group: *n* = 27 (female: 25), mean age 51.11 y.o.	Structural and functional connectivity between the amygdala and orbital frontal cortex via fMRI.	The degree of functional connectivity between the medial prefrontal cortex and the amygdala exhibited a positive correlation with the duration of illness in the BMS group.	The enhanced functional connectivity between the medial prefrontal cortex and amygdala, as observed in BMS patients, is associated with the severity of the disease.
Kurokawa et al., 2021 [13]	BMS group: *n* = 14 subjects, perimenopausal Control group: *n* = 11 age- and sex-matched healthy volunteers	Brain structural connectivity using probabilistic tractography and graph analysis.	The betweenness centrality exhibited a significant increase in the left insula, right amygdala, and right lateral orbitofrontal cortex, and a significant decrease in the right inferior temporal cortex in the BMS group compared to the healthy control group.	A graph analysis of brain probabilistic structural connectivity, based on diffusion imaging, revealed alterations in regions comprising the pain matrix and medial pain ascending pathway.
Kato et al., 2024 [14]	BMS group: *n* = 14 (female: 14), mean age 57.0 y.o. Control group: *n* = 11 (female: 11) healthy subjects, mean age 55.8 y.o.	Fractional anisotropy and volume fraction are used to examine the direction of neuronal connections.	In the BMS group, increased astrocyte volume and alterations in the microstructure of the amygdala were associated with pain.	The activation of emotional and affective neural circuits, along with structural and functional alterations, has been observed in patients diagnosed with BMS. These alterations are often associated with the presence of comorbid anxiety and depression.

Abbreviations: BMS: burning mouth syndrome; fMRI: functional magnetic resonance imaging; y.o.: years old.

## Data Availability

The data that support the findings of this study are available from the corresponding author upon reasonable request.

## Abbreviations

The following abbreviations are used in this manuscript:

ACC	Anterior cingulate cortex
AMY	Amygdala
BMS	Burning mouth syndrome
DTI	Diffusion tensor imaging
fMRI	Functional magnetic resonance imaging
Hc	Hippocampus
IC	Insular cortex
ICOP	International Classification of Orofacial Pain
mPFC	Medial prefrontal cortex
PET	Positron emission tomography
